# Conjugating uncoupler compounds with hydrophobic hydrocarbon chains to achieve adipose tissue selective drug accumulation

**DOI:** 10.1038/s41598-024-54466-2

**Published:** 2024-02-28

**Authors:** Mei Ying Ng, Zhi Jian Song, Gopalakrishnan Venkatesan, Sergio Rodriguez-Cuenca, James A. West, Shili Yang, Choon Hong Tan, Paul Chi-Lui Ho, Julian L. Griffin, Antonio Vidal-Puig, Marcella Bassetto, Thilo Hagen

**Affiliations:** 1 Department of Biochemistry, Yong Loo Lin School of Medicine, National University of Singapore, Singapore, Singapore; 2 School of Physical and Mathematical Sciences, Division of Chemistry and Biological Chemistry, Nanyang Technological University, Singapore, Singapore; 3 Department of Pharmacy, Faculty of Science, National University of Singapore, Singapore, Singapore; 4 Wellcome-MRC Institute of Metabolic Science and Medical Research Council Metabolic Diseases Unit, The University of Cambridge, Cambridge, UK; 5 Department of Biochemistry, The University of Cambridge, Cambridge, UK; 6 School of Pharmacy, Monash University Malaysia, 47500 Subang Jaya, Malaysia; 7 The Rowett Institute of Nutrition and Health, The University of Aberdeen, Aberdeen, UK; 8 School of Pharmacy and Pharmaceutical Sciences, College of Biomedical and Life Sciences, Cardiff University, Cardiff, UK; 9 Present Address: Department of Cancer Biology, Dana-Farber Cancer Institute, Boston, MA USA

**Keywords:** Endocrine system and metabolic diseases, Drug delivery, Medicinal chemistry

## Abstract

One potential approach for treating obesity is to increase energy expenditure in brown and white adipose tissue. Here we aimed to achieve this outcome by targeting mitochondrial uncoupler compounds selectively to adipose tissue, thus avoiding side effects from uncoupling in other tissues. Selective drug accumulation in adipose tissue has been observed with many lipophilic compounds and dyes. Hence, we explored the feasibility of conjugating uncoupler compounds with a lipophilic C8-hydrocarbon chain via an ether bond. We found that substituting the trifluoromethoxy group in the uncoupler FCCP with a C8-hydrocarbon chain resulted in potent uncoupling activity. Nonetheless, the compound did not elicit therapeutic effects in mice, likely as a consequence of metabolic instability resulting from rapid ether bond cleavage. A lipophilic analog of the uncoupler compound 2,6-dinitrophenol, in which a C8-hydrocarbon chain was conjugated via an ether bond in the para-position (2,6-dinitro-4-(octyloxy)phenol), exhibited increased uncoupling activity compared to the parent compound. However, in vivo pharmacokinetics studies suggested that 2,6-dinitro-4-(octyloxy)phenol was also metabolically unstable. In conclusion, conjugation of a hydrophobic hydrocarbon chain to uncoupler compounds resulted in sustained or improved uncoupling activity. However, an ether bond linkage led to metabolic instability, indicating the need to conjugate lipophilic groups via other chemical bonds.

## Introduction

Obesity is a major health problem with increasing prevalence in modern societies. It predisposes to a number of important chronic conditions including insulin resistance, type 2 diabetes mellitus (T2DM), nonalcoholic fatty liver disease, and cardiovascular diseases^1^. Obesity has also been identified as a risk factor for various malignancies^2^. Obesity is characterized by energy intake that exceeds energy expenditure. Consequently, the first-line treatment for obesity are lifestyle modifications to restrict caloric intake and increase physical activity. However, these interventions often fail to reliably sustain long-term weight loss^3^. Hence, pharmacotherapy using anti-obesity agents is one major cornerstone of effective obesity treatment.

The majority of currently FDA-approved prescription medications for weight loss work by decreasing energy intake via one of two mechanisms: a peripheral mechanism through inhibiting gastrointestinal lipase-mediated lipolysis to reduce the efficiency of intestinal lipid absorption or a central mechanism acting on neural circuits to suppress appetite and/or promote satiety^3–6^. However, these approaches have generally met with limited success primarily due to poor long-term clinical drug efficacy with only a subset of patients achieving clinically meaningful weight reduction^7–11^. Although promising results have been obtained recently in clinical trials with appetite-lowering Glucagon-like Peptide 1 (GLP-1) receptor agonists, the high cost of these therapies is currently a major limitation.

A general drawback with the strategy of reducing energy intake to achieve weight loss is its self-limiting homeostatic mechanism. The set-point theory posits that reduction in body weight induces feedback mechanisms that drive a compensatory decrease in basal metabolic rate and total energy expenditure, thus resulting in resistance to deviations from the body weight set point. This limits the maintenance of long-term weight loss with drugs that work via reducing energy intake^12–14^. Furthermore, prolonged use of these drugs has been associated with side effects, including orlistat-associated steatorrhoea, addictive and euphoric effects of amphetamines, as well as arrhythmogenic and hypertensive effects of adrenergic agents^15^. Therefore, alternative approaches are needed for effective obesity therapy.

One attractive alternative strategy to overcome these limitations is targeting energy expenditure. A highly effective approach to increase energy expenditure is through mitochondrial uncoupling. In normal cellular respiration, oxidation of fatty acids and glucose generate the high-energy electron carrier nicotinamide dinucleotide (NADH). Electrons derived from NADH oxidation by complex I of the electron transport chain are passed down the ETC complexes to the final electron acceptor molecular oxygen. In this process of electron transport, the free energy change is utilized to pump protons from the mitochondrial matrix into the intermembrane space against a concentration gradient. This generates a proton gradient across the inner mitochondrial membrane, which is then used by the F_0_F_1_-ATP synthase to synthesize ATP from ADP and inorganic phosphate. Continuous electron transport and substrate oxidation is dependent on availability of ADP and utilization of the transmembrane proton gradient. Under conditions of low cellular ATP utilization rates, ADP is maintained at low levels, resulting in accumulation of a high protein gradient across the inner membrane. This exerts protonic back pressure on the electron transport chain, leading to reduced rate of electron transport and hence substrate oxidation.

Small molecule mitochondrial uncoupler compounds uncouple electron transport and substrate oxidation from the availability of ADP. They function to shuttle protons from the mitochondrial intermembrane space into the matrix along the concentration gradient, resulting in dissipation of the mitochondrial proton gradient. Hence, uncouplers create futile cycles of substrate oxidation, leading to the conversion of the free energy change to heat without generating ATP, and increase energy expenditure^16^. The protonophoric function of uncouplers is due to their weakly acidic nature and ability to undergo reversible pH-dependent ionization as well as their capability to permeate the inner mitochondrial membrane in both their protonated and deprotonated form^16–19^.

One of the best-characterized chemical mitochondrial uncouplers is 2,4-dinitrophenol. This uncoupler compound was widely used as a highly effective weight loss-inducing drug in the 1930s. However, 2,4-dinitrophenol has a narrow therapeutic window and is associated with adverse side effects including hyperthermia, arrhythmia, and cataracts^20^. Importantly, these toxicities are largely due to on-target effects of systemic mitochondrial uncoupling, which result in uncontrolled heat dissipation, decreased ATP production, and increased cytosolic calcium concentration, potentially eventually leading to death. Furthermore, 2,4-dinitrophenol undergoes reduction to the metabolite 2-amino-4-nitrophenol, which is reported to be cataractogenic and is associated with DNP-induced blindness^21–24^. Due to the occurrences of these fatal adverse effects, the use of 2,4-dinitrophenol as an anti-obesity agent was suspended by the FDA and the drug taken off the market in 1938.

In recent years, there is a resurgence of interest in the use of mitochondrial uncoupling agents for obesity treatment^25^. This interest is driven by emerging evidence from a number of animal studies showing that with precise dosing, 2,4-dinitrophenol can confer beneficial effects in metabolic diseases including obesity and T2DM, as well as in lifespan extension^26,27^. Of note, a study by Perry et al.^28^ demonstrated that by targeting a derivative of 2,4-dinitrophenol to the liver, its therapeutic index could be significantly increased, resulting in reversal of hepatic hypertriglyceridemia, insulin resistance and fatty liver disease, without systemic adverse effects.

Recently, Liang et al.^29^ have pursued a novel approach to target 2,4-dinitrophenol to adipose tissue. The authors took advantage of the property of lipophilic compounds to selectively accumulate in adipose tissue and synthesized a tetradecanoic acid-2,4-dinitrophenol ester. The hydrophobic tetradecanoyl group was conjugated to the phenolic hydroxy of 2,4-dinitrophenol. Upon accumulation in adipocytes, the ester bond is cleaved by cellular esterases, giving rise to active 2,4-dinitrophenol. Experiments in high fat diet treated mice revealed that when tetradecanoic acid-2,4-dinitrophenol was administered orally or via a microneedle patch approach, the drug had similar or greater effects compared to 2,4-dinitrophenol on reducing body weight, body fat content, adipocyte size and fat content, serum content of triglyceride and low-density lipoprotein cholesterol, the respiratory quotient and fasting blood glucose levels. Importantly, conjugation of 2,4-dinitrophenol with a lipohilic hydrocarbon chain led to selective drug accumulation in adipose tissue and inhibited or prevented side effects associated with unconjugated 2,4-dintrophenol, such as an increase in several markers of cardiovascular risk and cardiac disease. This example highlights that an adipose tissue selective mitochondrial uncoupling approach may be beneficial in improving the safety profile of mitochondrial uncouplers.

In this study we took a different approach by synthesizing uncoupler compounds that are conjugated with aliphatic hydrocarbon chains that are not enzymatically hydrolyzed. We characterized the uncoupling activity of these lipophilic uncouplers as well as their functional and pharmacokinetic properties in vitro and in vivo.

## Results

### FCCP analogs with a hydrophobic hydrocarbon chain have uncoupling activity

**Figure 1:**
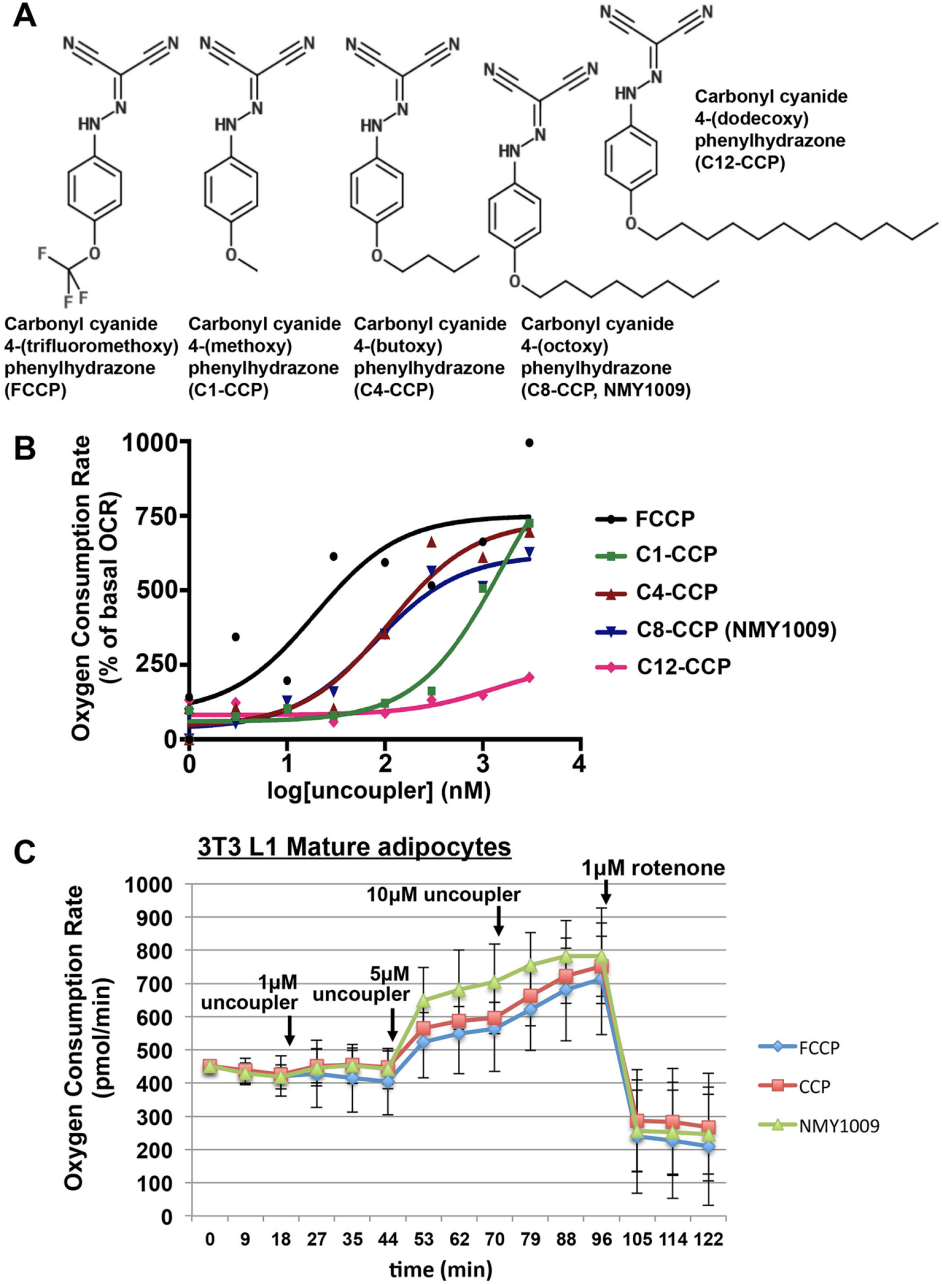
Uncoupling activity of hydrocarbon chain conjugated FCCP analogues. (**A**) Structure of the synthesized FCCP analogs. (**B**) The uncoupling activity of the various FCCP analogs was measured in isolated mouse liver mitochondria by first recording the basal respiratory rate in the presence of succinate and the ATP synthase inhibitor oligomycin A and then the uncoupled respiratory rate after the addition of different uncouplers at difference concentrations. The uncoupled respiratory rates were expressed as percent of the basal respiratory rate. (**C**) Differentiated 3T3 L1 adipocytes were seeded in a Seahorse 24-well plate and cellular oxygen consumption rates were monitored during the indicated time frame. The addition of different concentrations of uncoupler (FCCP, CCP or NMY1009) or rotenone is indicated.

To increase the lipophilicity of the mitochondrial uncoupler FCCP, we synthesized a number of analogs in which the trifluoromethoxy group was substituted with alkyl ether groups of different length (Fig. 1A). These substitutions have the potential to weaken the strong inductive electron-withdrawing properties of the trifluoromethoxy group and affect the pKa of the hydrazonyl dicyanide group, potentially resulting in lower uncoupling activity. We therefore compared the uncoupling activity of the various analogs. As shown in Fig. 1B, the analogs with C4- and C8-hydrocarbon chains exerted potent uncoupling activity in mouse liver mitochondria (EC50 of approximately 100 nM), although the compounds were slightly less potent compared to FCCP. C1-CCP, in which the trifluoromethoxy group was substituted with a methoxy group, exhibited lower uncoupling activity compared to C4-CCP and C8-CCP. Finally, the C12-hydrocarbon chain conjugated analog had no activity. Due to its activity and hydrophobicity, we hence selected the C8-hydrocarbon chain conjugated analog (which we will be referring to as NMY1009) in further studies.

Given the intended in vivo targeting of hydrocarbon chain-conjugated uncouplers to adipose tissue, we tested the uncoupling activity of NMY1009 in differentiated 3T3 L1 adipocytes using the Seahorse flux analyzer (Fig. 1C). NMY1009 exhibited a similar dose dependence in increasing basal oxygen consumption rates as FCCP and a FCCP analog lacking any functional group in the para-position (CCP).

### In vivo characterization of C8-hydrocarbon chain conjugated FCCP analog NMY1009

**Figure 2:**
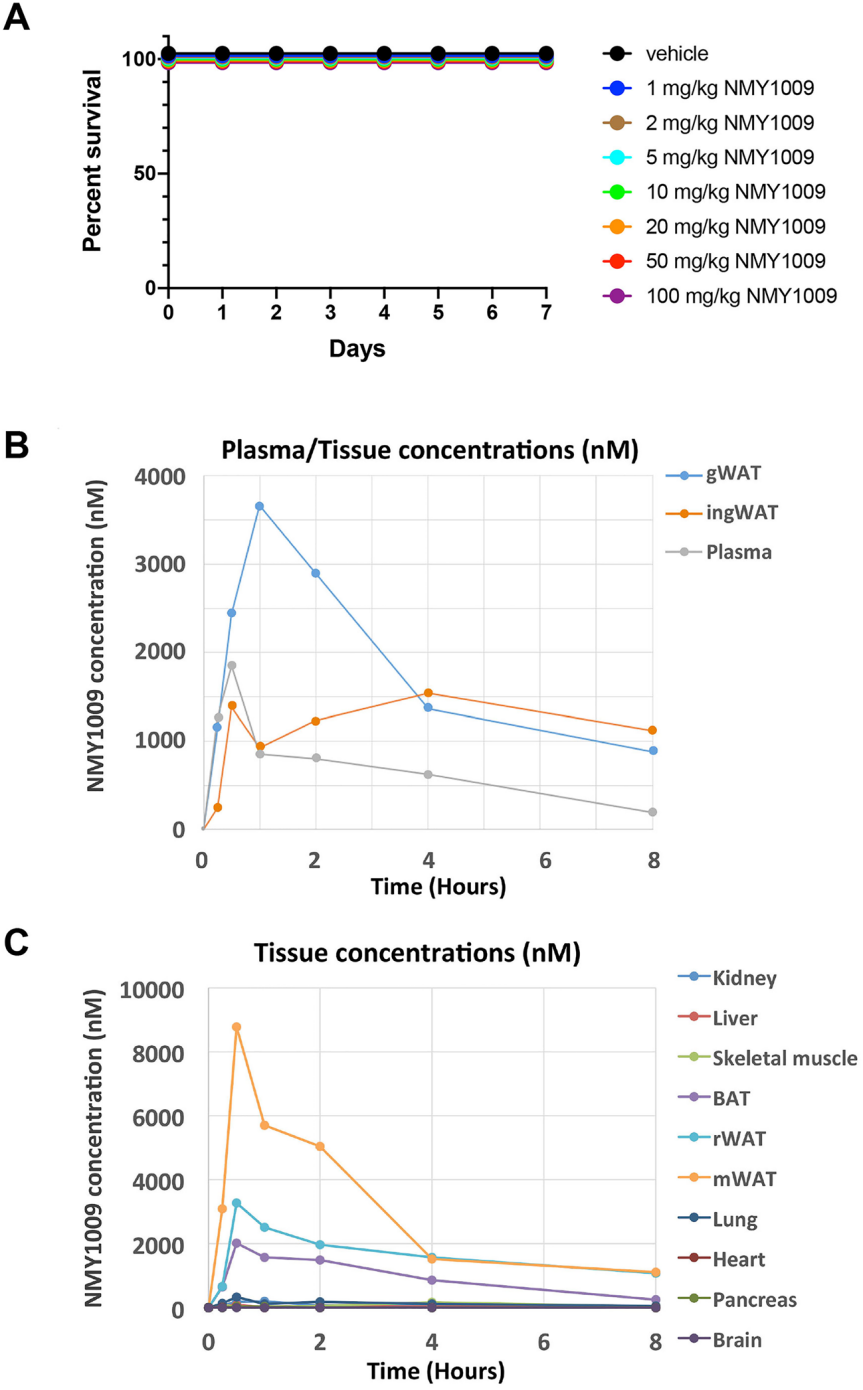
Determination of NMY1009 toxicity and plasma and tissue concentrations. (**A**) 7 treatment groups and 1 vehicle group (four mice per each group) were subjected to 7 daily administrations of the drug i.p. and well-being checks were carried out at 6 and 24 h after each treatment. No deaths or adverse reactions at any of the used NMY1009 doses were observed. (**B**, **C**) Tissue and plasma kinetics of NMY1009. Mice were injected with 5 mg/kg NMY1009 i.p. NMY1009 concentrations were measured by LC/MS.

To evaluate the in vivo effect of NMY1009, we initially conducted a toxicity study, in which mice were intraperitoneally injected daily with NMY1009 at doses from 1 to 100 mg/kg for seven days. No deaths or adverse reactions were observed (Fig. 2A and data not shown). NMY1009 was thus deemed safe for in vivo pharmacokinetic and functional studies.

To characterize the in vivo pharmacokinetics properties of NMY1009, we measured plasma and tissue concentrations over time after an i.p. injection of 5 mg/kg NMY1009. As shown in Fig. 2B, the NMY1009 plasma concentration peaked at 30 min and declined gradually thereafter. Notably, the highest drug tissue concentrations were found in the different types of adipose tissue, whereas all other tissues showed only very low drug concentrations (Fig. 2B,C). The kinetics of the tissue drug concentrations were similar compared to plasma. The observed decline in the adipose tissue concentrations may be due to drug metabolism or renal excretion. The latter possibility appears less likely as the drug concentration in the kidney was very low. In summary, the results suggest that NMY1009 accumulated selectively in adipose tissue. However, the adipose tissue drug concentrations decreased over time with a half-life of approximately 3 h.

**Figure 3:**
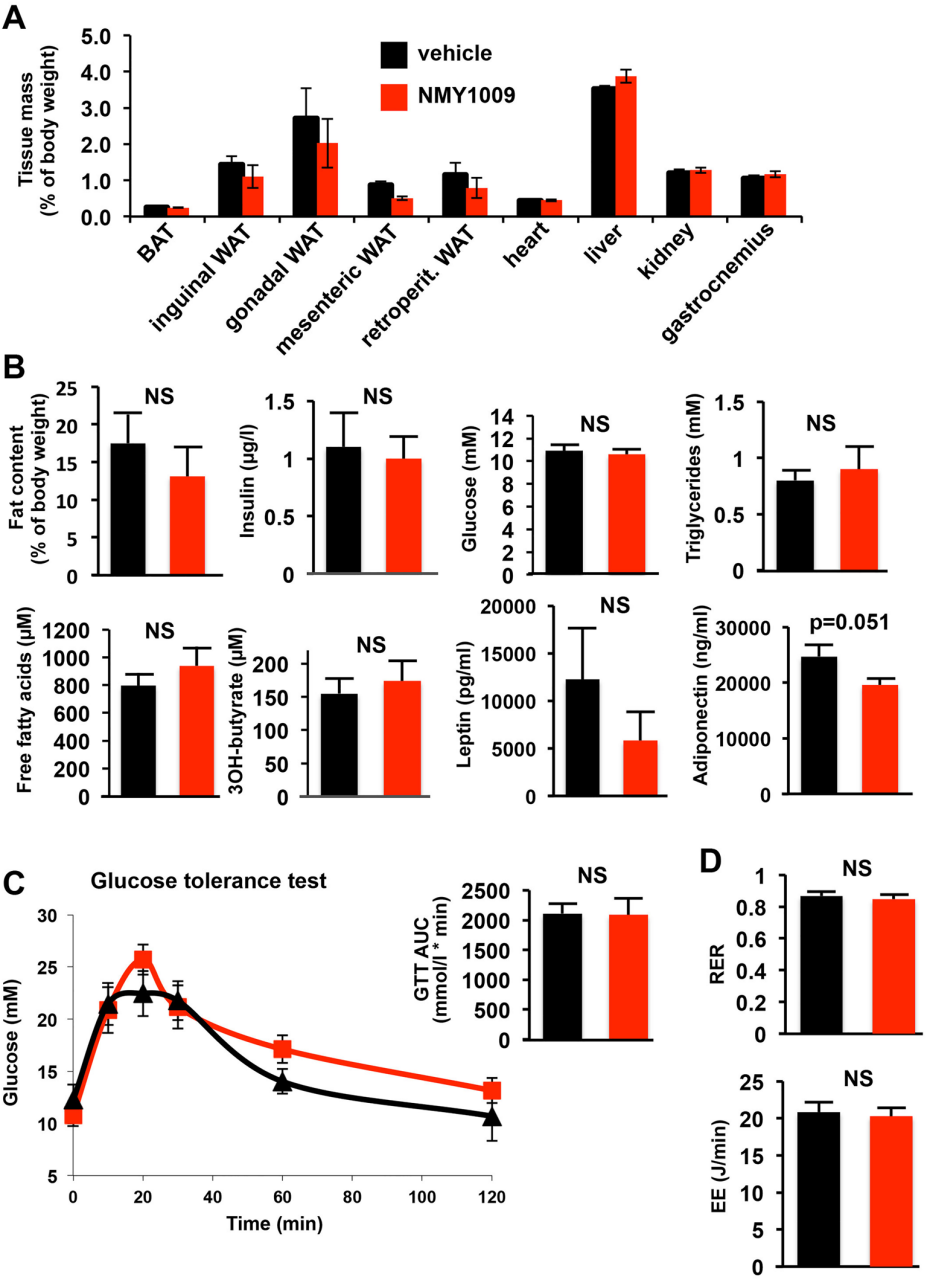
Functional mouse studies with NMY1009. Mice were subjected to 3 weeks of HFD treatment (45% fat content by calories with normal chow diet in the control group) with daily administration of 3 mg/kg NMY1009. (**A**, **B**) The body composition, fat content, blood glucose and serum insulin, triglyceride, free fatty acid, 3-hyroxybutyrate, leptin and adiponectin concentratons at the end of the 3 weeks treatment period were measured after culling of animals (n = 5 mice in each group). A second batch (n = 6 mice in each group) gave very similar results (data not shown). (**C**) The Glucose tolerance test (GTT) was performed at the end of the 3 weeks treatment (n = 5 in each group). There was no significant difference in the area under the curve (AUC) calculated over 120 min (2109 + 179 mmol/l × min for vehicle versus 2090 + 288 mmol/l x min for NMY1009), as shown in the bar graph. A second batch (n = 6 mice in each group) gave very similar results (data not shown). (**D**) Body oxygen consumption (VO_2_) and carbon dioxide production (VCO_2_) were measured by Oxymax Open Circuit Indirect Calorimetry. Respiratory exchange ratio (RER) and energy expenditure (EE) were calculated were as described previously (Lelliot 2006) Vehicle (n = 10), NMY1009 (n = 9).

Subsequently, functional mouse studies were conducted, in which mice were placed for three weeks on a high fat diet with daily i.p. administration of 3 mg/kg NMY1009. Compared to the vehicle treated control mice, there were no changes in the body composition (body weight and fat mass gains) (Fig. 3A) and no significant differences in blood glucose concentrations and other blood markers of metabolic health (with the exception of a decrease in serum adiponectin concentrations in NMY1009 treated mice; *p* = 0.051) (Fig. 3B). We also found no altered Glucose Tolerance Test response (Fig. 3C). Finally, Oxymax Open Circuit Indirect Calorimetry revealed no differences the respiratory exchange ratio and energy expenditure (Fig. 3D). The lack of functional effects was surprising, given various previous reports of beneficial effects of uncoupler treatment on body fat loss and metabolic markers.

### NM1009 is metabolically unstable

**Figure 4:**
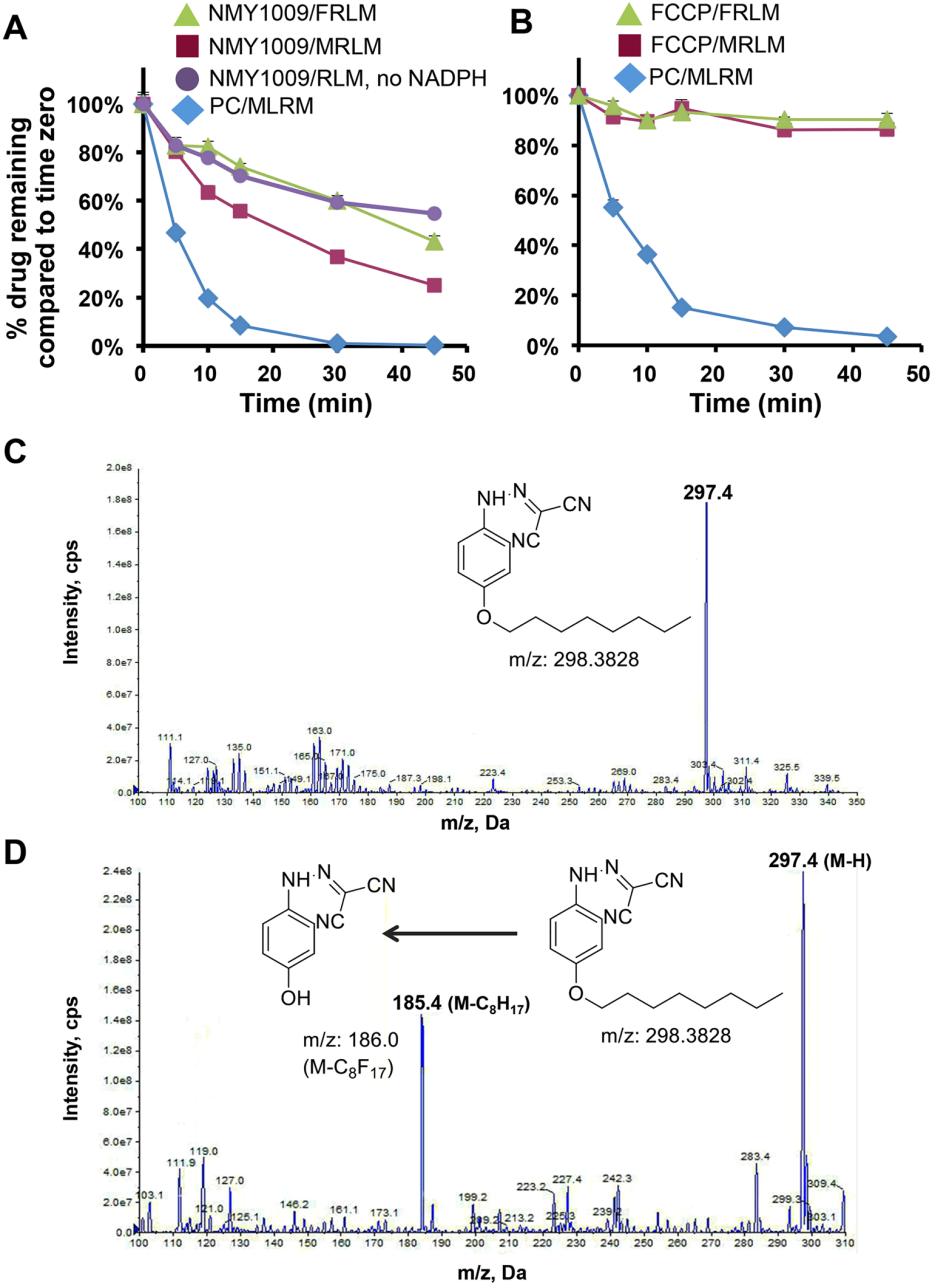
NMY1009 is unstable in aqueous solution. (**A**, **B**) Percentages of the remaining drug concentrations of NMY1009 (A) and FCCP (B) and the positive control midazolam (PC) relative to initial amounts (t = 0) upon incubation with female or male rat liver microsomes (FRLM and MRLM, respectively) at 37 °C for 5, 10, 15, 30 and 45 min. The percentages of remaining are expressed as the mean ± S.D. (n = 3). (**C**, **D**) NMY1009 is dealkylated in aqueous 100 mM KPO_4_ solution. (**C**) ESI (negative) spectra of NMY1009 samples at 0 min. (**D**) ESI (negative) spectra of NMY1009 samples at 1 h with the structure of the plausible degradation product of NMY1009 shown.

One potential explanation for the lack of functional effects of NMY1009 treatment is rapid drug metabolism. We hence evaluated the stability of NMY1009 upon incubation with rat liver microsomes. As shown in Fig. 4A, the concentration of NMY1009 decreased over time, with a drug half-life of approximately 30 min. Notably, similar drug turnover was observed in the absence of NADPH, suggesting that NMY1009 spontaneously degrades in aqueous solution. This result is markedly different from FCCP, which was found to be stable upon incubation with microsomes over the same time period in both the presence and absence of NADPH (Fig. 4B). Hence, these results suggest that the octoxy group in NMY1009 decreases the drug stability in aqueous solutions.

We subsequently performed ESI mass spectrometry studies after incubation of FCCP and NMY1009 in aqueous buffer to characterize the drug metabolism. In the ESI spectra of FCCP we observed a peak at 185.0 m/z in the negative ionization mode of ESI at the end of the 24 h incubation period. This peak corresponds to the O-dealkylated FCCP metabolite (loss of the trifluoromethyl group, data not shown). Notably, a dealkylated metabolite (loss of the octyl group) of NMY1009 was observed as early as 30 min (data not shown) and 1h (Fig. 4C,D) of the incubation in aqueous buffer. Hence, the results suggest that in aqueous solution, NMY1009 is subject to rapid O-dealkylation at the octoxy ether bond.

**Figure 5:**
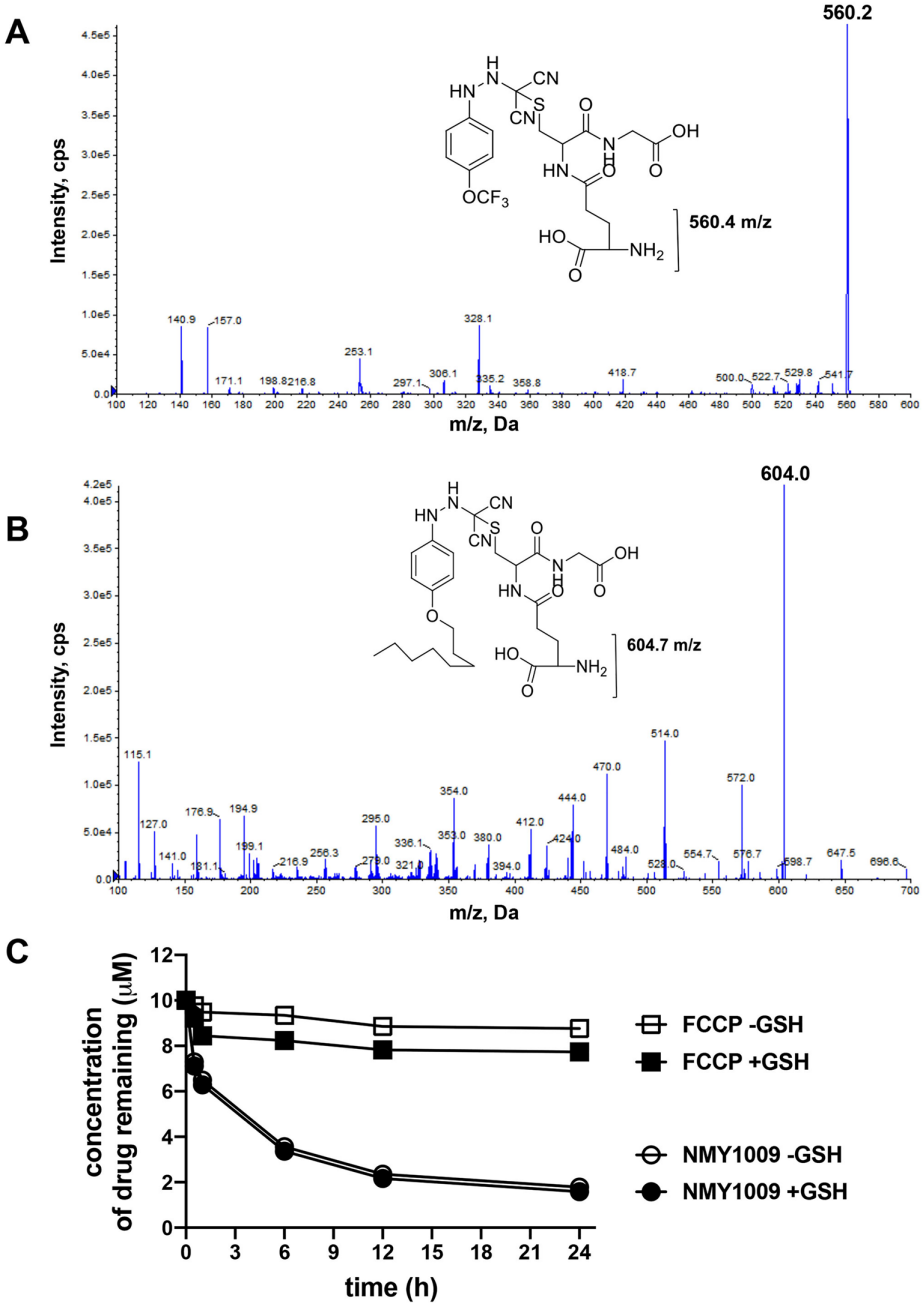
FCCP and NMY1009 form adducts with GSH but cysteine reactivity is unlikely to contribute to the rapid metabolism of NMY1009. (**A**) ESI–MS of FCCP-GSH adduct. (**B**) ESI–MS of NMY1009-GSH adduct. The incubation conditions for (A) and (B) were 10 μM NMY1009 or FCCP, 0.2 mM GSH in 100 mM KPO_4_ buffer, 1 h at 37 °C. (**C**) Percentages of the remaining NMY1009 (**A**) and FCCP after incubation in aqueous 100 mM KPO_4_ buffer in the presence or absence of GSH. The reaction mixtures contained 10 μM NMY1009 or FCCP in 100 mM KPO_4_ buffer with or without 0.2 mM GSH. The drug concentration were measured by LC–MS/MS. The incubations and drug concentration measurements were performed in triplicates and the shown results represent the means. Note that the error bars representing the S.D. values are covered by the symbols and too small to be seen.

FCCP has previously been reported to exhibit high reactivity with the thiol group in glutathione^30–32^. Glutathione is normally present at millimolar concentrations in cells. Hence, another possible cause of NMY1009 drug metabolism is that similar to FCCP, the compound exhibits reactivity with the reduced form of glutathione. To test this, FCCP and NMY1009 were incubated in the presence of reduced glutathione and adduct formation was determined by mass spectrometry. In the FCCP-GSH binding study we noticed a peak at 560.2 m/z in negative ionization mode of ESI-Mass spectra after incubation times of 30 min or 1 h (Fig. 5A). This peak corresponds to the formation of a covalent bond between the GSH thiol group and the tertiary carbon of the hydrazonyl dicyanide chain in FCCP. Similarly, a peak at 604.0 m/z was noticed in the negative ionization mode of ESI-Mass spectra of NMY1009, corresponding to the GSH adduct with NMY1009 (Fig. 5B). The results confirmed the presence of a strong electrophilic centre at the tertiary carbon for both FCCP and NMY1009, which reacts with nucleophilic GSH.

We then determined whether the reactivity with GSH may contribute to the uncoupler drug metabolism. To this end, we quantified the amount of FCCP and NMY1009 remaining over time during a 24 h incubation (37 **°**C) of the drugs in aqueous solution in the absence or presence of 0.2 mM GSH. Aliquots were removed at different time points and the remaining drug concentrations were determined. As shown in Fig. 5C, FCCP was not metabolized significantly over 24 h in the absence or presence of GSH. In contrast, NMY1009 was degraded over time in aqueous solution without GSH, with one third of the drug turned over after approximately 1 h and two thirds after 6h (Fig. 5C). At the end of the 24 h incubation, only about 18% of NMY 1009 remained. The drug turnover of NMY1009 was similar in the presence of GSH (Fig. 5C). In conclusion, while both FCCP and NMY1009 react with the thiol group in GSH, it is unlikely that the thiol reactivity of the uncoupler compounds contributes significantly to the drug metabolism. Thus, the main route of NMY1009 turnover is likely via spontaneous O-dealkylation in aqueous solution.

### Uncoupling activity and metabolic stability of 2,6-dinitro-4-(octyloxy)phenol

**Figure 6:**
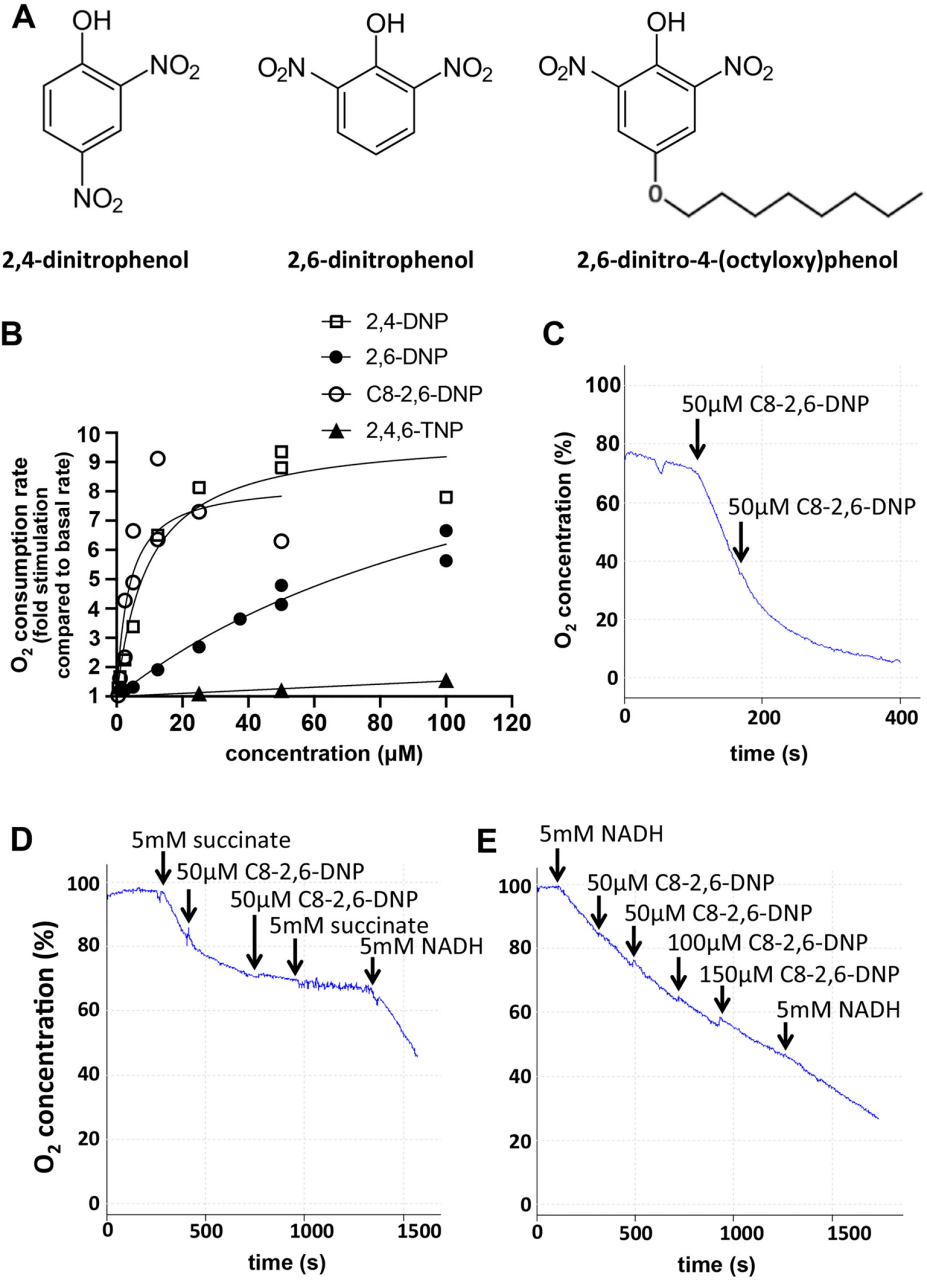
Characterization of the uncoupling activity of 2,6-dinitro-4-(octyloxy)phenol. (**A**) Structure of 2,4-dinitrophenol, 2,6-dinitrophenol and 2,6-dinitro-4-(octyloxy)phenol. (**B**) Dose response curve of the fold stimulation of oxygen consumption of isolated mouse liver mitochondria in the presence of succinate and oligomycin A after addition of increasing uncoupler concentrations. The following EC50 values were calculated using nonlinear fit in GraphPad Prism: 2,4-dinitrophenol (2,4-DNP): 9.6 μM; 2,6-dinitrophenol (2,6-DNP): 136 μM; 2,6-dinitro-4-(octyloxy)phenol (C8-2,6-DNP): 3.7 μM; 2,4,6-trinitrophenol (2,4,6-TNP): not applicable. (**C**) The oxygen consumption of isolated mouse liver mitochondria was measured in respiration buffer in the presence of succinate and oligomycin A with the indicated additions through the needle access port of the Clark electrode. (**D**,**E**) Isolated mouse liver mitochondria were subjected to a freeze–thaw cycle at − 20 °C and then incubated in the Clarke electrode chamber in respiration buffer. The addition of succinate or NADH respiratory substrate and 2,6-dinitro-4-(octyloxy)phenol (C8-2,6-DNP) are indicated.

We were interested to explore whether the instability of the octoxy ether bond is specific to NMY1009. Thus, as an alternative approach we synthesized a C8-hydrocarbon chain conjugated analogue of another uncoupler compound, 2,6-dinitrophenol, which is a less potent analogue of the widely used uncoupler 2,4-dinitrophenol. 2,6-dinitrophenol was conjugated with an octoxy group at the para position of the phenolic ring (2,6-dinitro-4-(octyloxy)phenol) (Fig. 6A).

We first explored the effect of the hydrocarbon chain conjugation on the uncoupling activity in comparison to 2,4-dinitrophenol, 2,6-dinitrophenol and 2,4,6-trinitrophenol. Consistent with previous reports, 2,4-dinitrophenol was the most potent uncoupler (Fig. 6B). 2,6-dinitrophenol had lower activity. 2,4,6-trinitrophenol exhibited no significant uncoupling activity, as previously reported^33^, and this is likely due to the low pKa of 0.25 of the phenolic hydroxy group in 2,4,6-trinitrophenol. Interestingly, 2,6-dinitro-4-(octyloxy)phenol was markedly more potent in uncoupling isolated mitochondria compared to 2,6-dinitrophenol and showed a similar dose response compared to 2,4-dinitrophenol. These results indicate that the octoxy group promotes the uncoupling activity, which may be due to a change in membrane partitioning or lowering of the pKa value of the hydroxy group.

We also noted that unlike 2,4-dinitrophenol and 2,6-dinitrophenol, 2,6-dinitro-4-(octyloxy)phenol inhibited mitochondrial oxygen consumption at very high concentrations in both isolated mitochondria and intact cells (Fig. 6C and data not shown). In contrast, 2,4-dinitrophenol exhibited no inhibitory effect on mitochondrial electron transport chain activity at concentrations up to 350 µM (data not shown). 2,6-dinitro-4-(octyloxy)phenol also inhibited succinate-dependent oxygen consumption of mouse liver mitochondria after disruption of the inner membrane (Fig. 6D), indicating that the effect of the drug is due to a direct effect on the electron transport chain. In contrast to succinate supported respiration, 2,6-dinitro-4-(octyloxy)phenol only had a minimal effect when NADH was used as a substrate (Fig. 6E). These results suggest that the inhibitory effect of 2,6-dinitro-4-(octyloxy)phenol is due to a direct effect on complex II. Consistent with this, addition of reducing agent ascorbate and TMPD, which donates electrons to cytochrome c, rescued the inhibitory effect of 2,6-dinitro-4-(octyloxy)phenol on succinate dependent respiration in both intact and disrupted mitochondria (data not shown).

**Figure 7:**
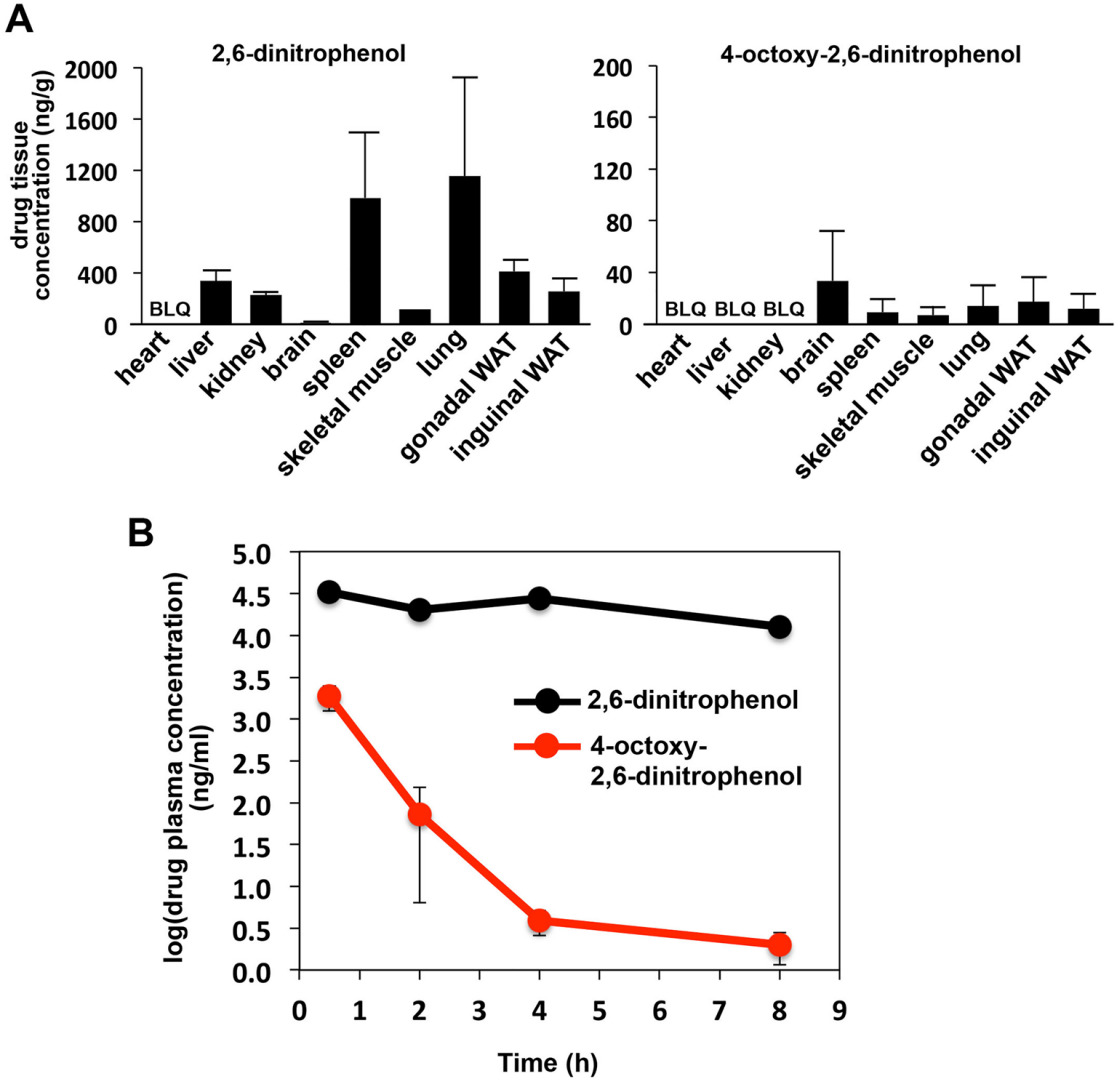
Pharmacokinetics of 2,6-dinitro-4-(octyloxy)phenol. (**A**) 5 mg/kg 2,6-dinitrophenol or 2,6-dinitro-4-(octyloxy)phenol in 40% (2-Hydroxypropyl)-β-cyclodextrin was administered to three male C57BL/6 mice per group. After two hours, the mice were sacrificed and the indicated tissues were collected. The drug tissue concentrations were measured as described in Materials and Methods. The indicated concentrations correspond to ng of drug analog/g of wet tissue weight (means with standard deviation from n = 3; BLQ = Below Limit of Quantification.) (**B**) To measure plasma drug concentration kinetics, three mice per group were injected with a single dose of 5 mg/kg 2,6-dinitrophenol or 2,6-dinitro-4-(octyloxy)phenol. At time points 30 min, 2 h, 4 h and 8 h, mice were sacrificed and blood was collected. The drug concentrations were quantified as described under Materials and Methods (means with standard deviation from n = 3). All animals were found to be healthy throughout the study duration.

To characterize 2,6-dinitro-4-(octyloxy)phenol in vivo pharmacokinetics, we measured the drug tissue and plasma concentrations upon intraperitoneally injecting 2,6-dinitro-4-(octyloxy)phenol and the parent drug 2,6-dinitrophenol into mice at a dose of 5 mg/kg. As shown in Fig. 7A, 2,6-dinitrophenol exhibited a heterogeneous drug accumulation in the various tissues, with the highest concentrations found in the spleen and lung. Compared to 2,6-dinitrophenol, the detected tissue concentrations of 2,6-dinitro-4-(octyloxy)phenol were very low, suggesting that the drug was unstable. Consistent with this, the plasma concentration of 2,6-dinitro-4-(octyloxy)phenol decreased rapidly with much faster kinetics compared to 2,6-dinitrophenol (Fig. 7B). In conclusion, our results suggest that similar to FCCP, conjugation of 2,6-dinitrophenol with a C8-hydrocarbon chain via an ether bond results in sustained uncoupling activity, but leads to drug instability. Other approaches to attach lipophilic moieties to uncoupler compounds should be explored in the future.

## Discussion

In this study, we explored the feasibility of targeting uncoupler compounds to adipose tissue through conjugation of a hydrophobic hydrocarbon chain via an ether bond. The octoxy analogue of FCCP, NMY1009, as well as that of 2,6-dinitrophenol (2,6-dinitro-4-(octyloxy)phenol), exhibited potent uncoupling activity similar or superior to their parent compounds. In the case of NMY1009, adipose tissue selective drug accumulation was also achieved. However, both NMY1009 and 2,6-dinitro-4-(octyloxy)phenol exhibited metabolic instability in vivo.

Our approach to target uncouplers selectively to adipose tissue is based on the well-known characteristic of lipid soluble chemicals to be bioconcentrated in animal fat depots^34–36^. This would be expected to result in a relatively high steady state concentration in adipocytes and long-lasting therapeutic effects.

Another potentially favorable characteristic of lipid modified uncoupler compounds is their high affinity for biological membranes, resulting in greater cellular retention and sustained release of the uncouplers from membranes to elicit mitochondrial effects. In this regard, Morstein et al.^37^ have highlighted the favorable properties of C10-hydrocarbon chain modified compounds, compared to conjugation with hydrocarbon chains of shorter (C2) or greater (C14 and C18) length. On the one hand, C10-hydrocarbon chain conjugated compounds have a greater ability to partition into the lipid bilayer compared to C2 hydrocarbon chain compounds. On the other hand, C10-hydrocarbon chain modification results in a less negative partition free energy and consequently more ready release from membranes into the intracellular regions compared to C18-hydrocarbon chain conjugation. It appears likely that similar to the C10-hydrocarbon chain compounds described by Morstein et al.^37^, the C8-hydrocarbon chain conjugated compounds used in our study are characterized by a good lipophilicity with ready release into the cell. This may, at least in part, explain the potent uncoupling activity of these compounds. On the other hand, a high partition free energy may be responsible for the low uncoupling activity of the C12-CCP compound used in our study.

As recognized in a number of previous studies, uncoupler compounds modified with hydrocarbon chain of different lengths exhibit good uncoupling activities^18,38–40^, in some cases even higher than their parent compounds. For instance, Hemker^18^ reported that conjugation of 2,6-dinitrophenol with a 4-isooctyl group increased the uncoupling activity by one order of magnitude. We found that modification of 2,6-dinitrophenol with a 4-octoxy group decreased the EC50 value for stimulation of mitochondrial oxygen consumption from 136 μM (2,6-dinitrophenol) to 3.7 μM (2,6-dinitro-4-(octyloxy)phenol), indicating a marked increase in uncoupling activity.

Similarly, C8-hydrocarbon chain conjugated CCP (NMY1009) was more potent in promoting mitochondrial uncoupling compared to conjugation with a C1-hydrocarbon chain (C1-CCP). In contrast, NMY1009 was not more potent compared to FCCP, which contains a 4-trifluoromethoxy group instead of a methoxy or octoxy group. The trifluoromethoxy group likely functions by stabilizing the anionic form of the hydrazone group, which mediates the uncoupling activity, via long range electron withdrawing effects^41^. The electron withdrawing property of the trifluoromethoxy group are due to both inductive and resonance effects. In contrast, the 4-methoxy group is less anion-stabilizing as it is less electronegative and hence exerts less inductive electron-withdrawing effects^42^. Moreover, the inductive electron-withdrawing effects are countered by the ability of the methoxy group to donate electrons to the benzene ring via resonance.

Compared to the methoxy group, the octoxy group exhibits a lower electronegativity and exerts even less electron-withdrawing and anion-stabilizing effects on the hydrazone group. Hence, there must be other reasons for the improved uncoupling activity of NMY1009 compared to C1-CCP. These may include better plasma membrane permeation, an improved ability to permeate the inner mitochondria membrane in both the protonated and deprotonated state and a more favourable lipid membrane partitioning of NMY1009, resulting in greater inner mitochondrial membrane accumulation and sustained release of NMY1009 into the mitochondrial matrix and intermembrane space.

NMY1009 at concentrations up to 100 mg/kg exerted no toxicity over a seven day treatment period. In contrast, FCCP was found in a recent study that compared the toxicity of a number of uncouplers to cause adverse effects in rats at doses of 5mg/kg and higher, including death at doses higher than 20 mg/kg^43^. It is unclear whether the observed toxic effects of FCCP were due to uncoupling activity or off-target effects. There are a number of potential explanations for the different toxicity profiles of NMY1009 and FCCP, including species differences and NM1009 drug instability (see below). However, it is likely that even after dealkylation the degradation product of NMY1009 retains at least some uncoupling activity. Finally, it is possible that FCCP, via its trifluoromethoxy group, exerts greater off-target effects, which could be due to uncoupling-independent effects or dissipation of the proton gradient and membrane potential of other cellular membranes, such as the plasma or the lysosomal membrane. NMY1009 may be more selective towards mitochondria as a consequence of a higher pKa value of the proton-donating hydrazine group, resulting in selective deprotonation in the more alkaline mitochondrial matrix space.

Despite their good uncoupling activities, both NMY1009 and 2,6-dinitro-4-(octyloxy)phenol were found to be unstable in vivo. It has been reported previously^31^ and confirmed in our study that FCCP reacts with cysteines, e.g. in glutathione, via its hydrazine group, and that high concentrations of FCCP can induce the depletion of cellular glutathione pools^31,44,45^. Thiol reactivity of FCCP is likely mediated via its strong electrophilic centre at the tertiary carbon in the hydrazone group. NMY1009, which shares its hydrazone group with FCCP, was found to exhibit a similar reactivity with glutathione (Fig. 5B). However, the metabolic instability of NMY1009 is unlikely a consequence of reaction with thiols because the reaction rate of cysteine adduct formation is slow. Furthermore, unlike NMY1009, FCCP did not undergo significant degradation over 24 h, despite reacting with thiol groups.

In further metabolic stability mass spectrometry studies we found that NMY1009 is subject to rapid O-dealkylation (i.e. loss of the octyl group) in aqueous solution. A potential explanation is that the hydrazine group functions as a strong activating group of the benzene ring. It thus increases the reactivity at the ortho and para positioned substitution groups through resonance electron transfer through the benzene system. Consequently, the hydrazone group promotes the O-dealkylation reaction of NMY1009 at the para position.

We also obtained evidence that similar to NMY1009, 2,6-dinitro-4-(octyloxy)phenol is metabolically unstable. Given the much greater stability of 2,6-dinitrophenol, we hypothesize that 2,6-dinitro-4-(octyloxy)phenol also undergoes rapid O-dealkylation as a consequence of the meta-directing effects of the 2- and 6-nitro groups, although we have not formally confirmed this.

Despite the metabolic instability of the lipid-modified uncouplers synthesized in this study, we believe that there is great potential in the approach to target uncoupler drugs to adipose tissue, and that our study provides important insights into how this outcome can be achieved. While Liang et al.^29^ reported that conjugation of 2,4-dinitrophenol with a hydrocarbon chain via a reversible ester bond with the phenolic hydroxy is a promising approach, reversibility of the lipid modification is not necessary. Thus, we found that dinitrophenol- and FCCP-related uncouplers can uncouple well in the presence of an 8C-hydrocarbon chain. To avoid the rapid in vivo cleavage of the octoxy ether group, the 8C-hydrocarbon chain could be conjugated to the phenolic ring directly via a C–C linkage.

An important proof of concept for targeting mitochondrial uncouplers selectively to adipose tissue has been provided by Kopecky et al.^46^. The authors selectively overexpressed UCP1 ectopically in white adipose tissue under control of the adipocyte lipid-binding protein aP2 gene promoter. Transgenic mice with adipocyte-specific overexpression of UCP1 displayed significant reductions in total body weight and subcutaneous fat stores in both genetic and dietary-induced obesity^47,48^. Furthermore, this effect was later described to be partly a consequence of long-term changes involving induction of mitochondrial biogenesis in the white adipose tissue^47^. These findings suggest yet another benefit of prolonged uncoupling with adipose selective uncouplers, induction of mitochondrial biogenesis and adipose tissue browning. As a consequence, life style based interventions such as exercise, which increases β-adrenergic stimulation of adipose tissue and induces increased adipose tissue energy expenditure, may potentiate the therapeutic effects of mitochondrial uncoupling.

## Materials and methods

### Materials

2,4-dinitrophenol (DNP) and carbonyl cyanide *p*-trifluoromethoxy-phenylhydrazone (FCCP) were obtained from Sigma-Aldrich. 2,6-dinitrophenol was obtained from Aurum Pharmatech.

### Synthesis of 2,6-dinitro-4-(octyloxy)phenol

**Figure 8:**
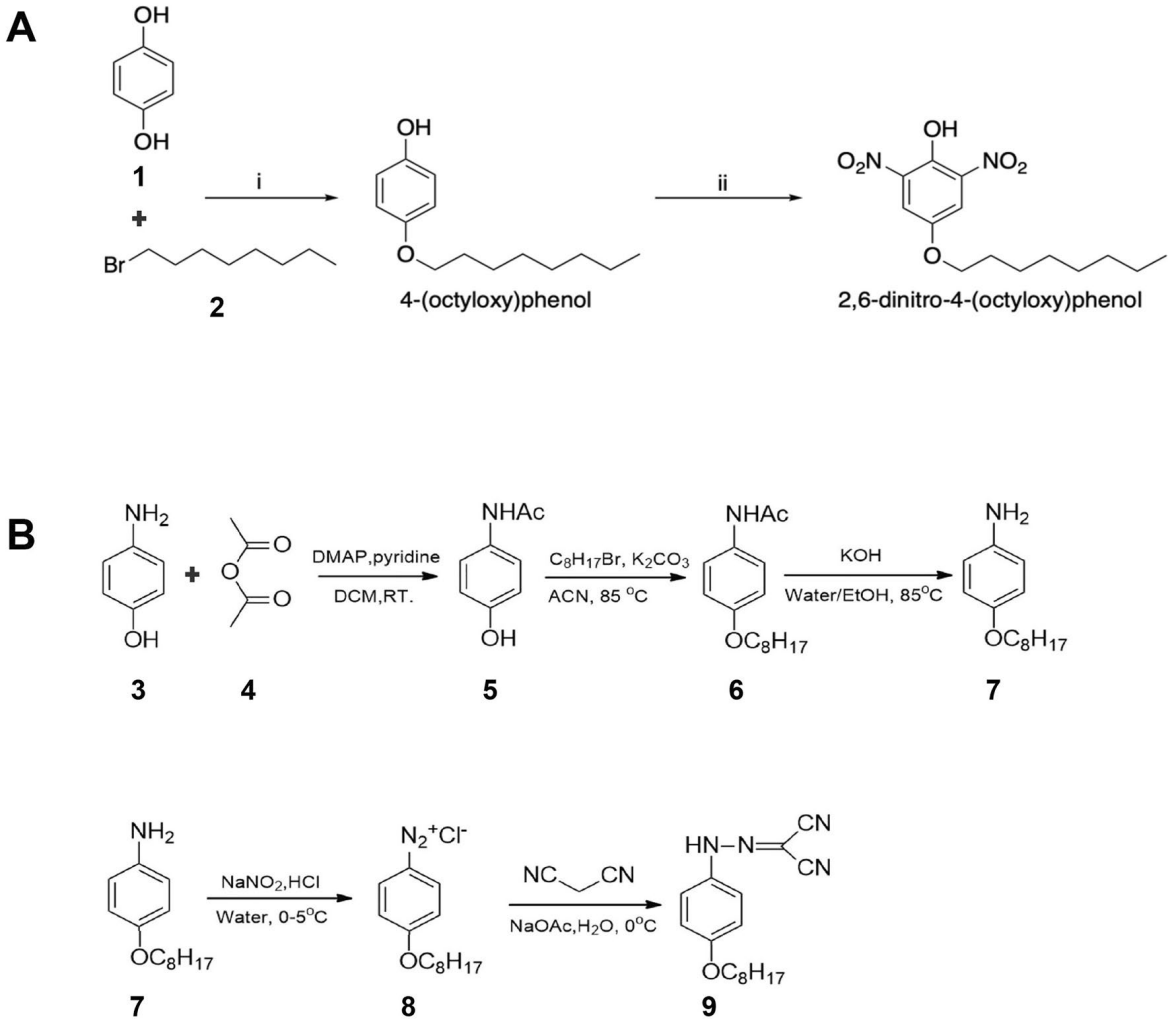
Synthesis of 2,6-dinitro-4-(octyloxy)phenol and NMY1009. (**A**) Synthesis of 2,6-dinitro-4-(octyloxy)phenol. *Reagents and conditions:* i. K_2_CO_3_, anhydrous DMF, 80 °C, o.n. (87%); ii. trichloroisocyanuric acid, wet SiO_2_ (50% w/w), NaNO_2_, r.t., 30 min. (28%). (**B**) Synthesis of (4-octyloxy-phenyl)carbonhydrazonoyl dicyanide (NMY1009).

2,6-Dinitro-4-(octyloxy)phenol was prepared according to a two-step synthesis, starting with the alkylation of hydroquinone **1** with 1-bromooctane **2**, followed by the di-nitration of the 4-(octyloxy)phenol intermediate with trichloroisocyanuric acid and NaNO_2_ (Fig. 8A), following a previously reported procedure^49^.

#### Synthetic chemistry methods

All solvents and reagents were used as obtained from commercial sources unless otherwise indicated. All solvents used for chromatography were HPLC grade from Fisher Scientific (UK). All reactions were performed under a nitrogen atmosphere. ^1^H and ^13^C-NMR spectra were recorded with a Bruker Avance III HD spectrometer operating at 500 MHz for ^1^H and 125 MHz for ^13^C, with Me_4_Si as internal standard. Deuterated chloroform was used as the solvent for NMR experiments, unless otherwise stated. ^1^H chemical shifts values (δ) are referenced to the residual non-deuterated components of the NMR solvents (δ = 7.26 ppm for CHCl_3_, etc.). The ^13^C chemical shifts (δ) are referenced to CDCl_3_ (central peak, δ = 77.0 ppm). Thin Layer Chromatography was performed on silica gel 60 F254 plastic sheets. Flash column chromatography was performed using silica cartridges in a Biotage Isolera automated system. UPLC-MS analysis was conducted on a Waters UPLC system with both Diode Array detection and Electrospray (+ ’ve and – ‘ve ionization) MS detection. The stationary phase was a Waters Acquity UPLC BEH C18 1.7um 2.1 × 50mm column. The mobile phase was LC–MS grade H_2_O containing 0.1% formic acid (A) and LC–MS grade MeCN containing 0.1% formic acid (B). Column temperature: 40 °C. Sample diluent: MeCN. Sample concentration 1µg/mL. Injection volume 2 µL. A linear gradient standard method was used: 90% A (0.1 min), 90%-0% A (2.1 min), 0% A (0.8 min), 90% A (0.1 min); flow rate 0.5 mL/min.

#### Synthesis of 2,6-dinitro-4-(octyloxy)phenol (Fig. 8A)

Step 1. Hydroquinone **1** (1.92 g, 17.4 mmol) and K_2_CO_3_ (2.40 g, 17.4 mmol) were suspended in anhydrous DMF (7.5 mL) under a nitrogen atmosphere. 1-Bromooctane **2** (1 mL, 5.8 mmol) was then added dropwise to the mixture, and the reaction was stirred at 80 °C overnight. The mixture was then cooled to room temperature, added of 10% aqueous HCl solution (10 mL), and extracted with DCM (3 × 20 mL). The combined organic layers were dried over Na_2_SO_4_ and concentrated under *vacuum*. The crude residue was purified by automated flash column chromatography (Biotage Isolera One, SNAP KP Sil 25 g) eluting with *n*-hexane:DCM 100:0 v/v increasing to 0:100 v/v in 10 CV, to give intermediate 4-(octyloxy)phenol as a pale yellow waxy solid in 87% yield.^1^H-NMR (500 MHz CDCl_3_), δ: 6.78 (d, J = 9.2 Hz, 2H), 6.74 (d, J = 9.2 Hz, 2H), 4.50 (s, 1H), 3.89 (t, J = 6.6 Hz, 2H), 1.77–1.72 (m, 2H), 1.46–1.40 (m, 2H), 1.37–1.25 (m, 8H), 0.88 (t, J = 7.1 Hz, 3H).

Step 2. A mixture of 4-(octyloxy)phenol (0.25 g, 1.12 mmol), trichloroisocyanuric acid (0.26 g, 1.12 mmol), NaNO_2_ (0.15 g, 2.24 mmol) and wet SiO_2_ (1g, 50% w/w) was shaken at room temperature for 30 min. The reaction was then added to DCM (20 mL) and filtered under *vacuum*. The filtrate was dried over Na_2_SO_4_ and concentrated to dryness. The crude residue was purified by automated flash column chromatography (Biotage Isolera One, SNAP KP Sil 25 g) eluting with *n*-hexane:DCM 100:0 v/v increasing to 0:100 v/v in 12 CV, to give the title compound as a yellow oil in 28% yield. ^1^H-NMR (500 MHz, CDCl_3_), δ: 11.00 (bs, 1H), 7.84 (s, 2H), 4.01 (t, J = 6.4 Hz, 2H), 1.84–1.77 (m, 2H), 1.48–1.41 (m, 2H), 1.37–1.23 (m, 8H), 0.89 (t, J = 7.1 Hz, 3H). ^13^C-NMR (125 MHz, CDCl_3_), δ: 150.3, 143.5, 137.5, 117.4, 69.7, 31.7, 29.3, 29.2, 29.1, 25.8, 22.6, 14.1. UPLC-MS: R_t_ 2.39 min, MS [ESI, m/z]: 311.1 [M-H]. Compounds 4-(octyloxy)phenol and 2,6-dinitro-4-(octyloxy)phenol were > 95% pure according to the UPLC-MS experiments performed.

### Synthesis of NMY1009

NMY1009 ((4-octyloxy-phenyl)carbonhydrazonoyl dicyanide, **9**) was synthesized using 4-Aminophenol (**3**) as starting material, as shown in Fig. 8B. 4-Aminophenol (**3,** 2.18 g) was added to a round bottom flask with 50 ml dry dichloromethane and pyridine (1.9 ml). 2 ml acetic anhydride was added to the mixture while slowly with stirring at room temperature. 4-Dimethylaminopyridine (240 mg) was added to the reaction under continuous stirring in a N_2_ protected atmosphere for 8 h. The product **5** was collected after flash column chromatography to obtain white solid with 90% yield. ^1^H NMR (400 MHz, MeOD) δ 7.40–7.26 (m, 2H), 6.81- 6.68 (m, 2H), 2.11 (s, 3H). MS [m/z]: 151.06, detected [ESI + , M + H^+^] 152.04.

N-(4-hydroxyphenyl)acetamide (**5**, 1.51 g), 1-bromooctane (2.89 g), and K_2_CO_3_ (4.14 g) was added to dry acetonitrile (100ml), the mixture was refluxed for 24 h with N_2_ protection and 3 g product **6** was collected after extraction and flash column chromatography. ^1^H NMR (400 MHz,) δ 7.41–7.33 (m, 2H), 7.17 (s, 1H), 6.87–6.81 (m, 2H), 3.92 (t, *J* = 6.6 Hz, 2H), 2.14 (s, 3H), 1.80–1.71 (m, 2H), 1.49–1.39 (m, 2H), 1.36–1.24 (m, 8H), 0.88 (t, *J* = 6.8 Hz, 3H). MS [m/z]: 263.19, deteced [ESI + , M + H^+^] 264.27.

N-(4-(octyloxy)phenyl)acetamide (**6**, 1 g) and KOH (4 g) were added to Ethanol/H_2_O (50 ml/10 ml) mixed solvent. The reaction was refluxed at 85 °C overnight. 760 mg (90% yield) oil like compound **7** was obtained after purification. ^1^H NMR (400 MHz,) δ 6.79–6.73 (m, 2H), 6.67–6.62 (m, 2H), 3.89 (t, *J* = 6.6 Hz, 2H), 1.75 (dt, *J* = 14.6, 6.7 Hz, 2H), 1.51–1.36 (m, 2H), 1.31 (ddd, *J* = 15.2, 6.7, 4.2 Hz, 8H), 0.90 (t, *J* = 6.9 Hz, 3H). MS [m/z]: 221.18, detected [ESI + , M + H^+^] 222.17.

NMY1009 (**9**) was synthesized via a one-step reaction by using 4-(octyloxy)aniline (**7)**. 4-(octyloxy)aniline (**7**, 610.7 mg) and concentrated HCl (2.5 ml) were added to 17 ml deionized water and then cooled to 0 °C to obtain a suspension. NaNO_2_ (190 mg) was dissolved in 1 ml water and then added to the aniline mixture with stirring to obtain the intermediate compound **8** (Clear solution). Malononitrile (270 mg) and sodium acetate (6.9 g) were added to 30ml deionized water and cooled to 0 °C with stirring. The intermediate **8** was added dropwise to the malononitrile solution. The reaction mixture was precipitated followed by filtration and washing and then dried under vacuum. NMY1009 (**9**) was obtained with 85% yield as a yellow solid. ^1^H NMR (400 MHz, CDCl_3_) δ 10.09 (s, 1H), 7.33–7.24 (m, 2H), 7.00–6.89 (m, 2H), 3.98 (t, *J* = 6.5 Hz, 2H), 1.86–1.75 (m, 2H), 1.54–1.42 (m, 2H), 1.42–1.26 (m, 8H), 0.91 (t, *J* = 6.8 Hz, 3H). ^13^C NMR (100 MHz, CDCl_3_) δ 158.18, 133.38, 129.29, 117.57, 115.63, 114.51, 112.78, 109.03, 84.65, 68.53, 31.81, 29.33, 29.22, 29.18, 26.00, 22.66, 14.10. MS [m/z]: 298.18, detected [ESI-, M-H^-^]: 297.25. The synthesis of the other (4-alkoxy-phenyl)carbonhydrazonoyl dicyanide compounds was carried out in an analogous manner.

### Isolation of mouse liver mitochondria and oxygen consumption measurements

Liver tissue was obtained from male C57BL/6JINV (Jax) mice. The mice were maintained using protocols approved by the Institutional Animal Care and Use Committee (IACUC) of National University of Singapore. The mice were euthanized by cervical dislocation under deep anesthesia with 5% isoflurane. Mouse liver mitochondria were isolated by homogenization and differential centrifugation at 4 °C in mitochondria isolation buffer (280 mM sucrose, 10 mM Tris–HCl, 1 mM EDTA (pH 7.4 at 4 °C)). In brief, minced tissue was homogenized in mitochondria isolation buffer before centrifugation to pellet the nuclei (700xg, 5 min, 4 °C). The resulting supernatant was centrifuged (9,000xg, 5 min, 4 °C) to pellet the mitochondria, followed by two washes and resuspending of the final mitochondrial pellet in mitochondrial isolation buffer.

Oxygen consumption of mouse liver mitochondria was measured in respiration buffer (280 mM sucrose, 10 mM Tris (pH7.4), 1 mM EDTA, 2.5 mM potassium phosphate and 2.5 mM MgCl_2_) at a mitochondrial protein concentration of 0.5 mg/ml and a temperature of 30 **°**C.

### Measuring cellular respiration in differentiated 3T3 L1 adipocytes using the Seahorse Flux Analyzer

To generate 3T3 L1 mature adipocytes, 3T3 L1 preadipocytes were plated at 6000 cells per well in DMEM supplemented with 10% newborn calf serum. After 4 days, cells were induced to differentiate with high glucose (4.5 g/l) DMEM supplemented with 10% fetal bovine serum (FBS), 5 μg/ml insulin, 1 μM dexamethasone and 0.5 mM 3-isobutyl-1-methylxanthine (IBMX). After 3 days, the induction cocktail was replaced with FBS supplemented DMEM containing 5 μg/ml insulin. Thereafter, cells were maintained in fresh FBS-supplemented DMEM every 3 days. Day nine differentiated 3T3 L1 mature adipocytes were assayed for cellular respiration rates.

Cells were plated at 15,000 cells per well in a 24-well Seahorse plate (Seahorse Bioscience) in complete growth medium and adhered overnight in a humidified 37 °C, 5% CO_2_ incubator. A sensor cartridge was hydrated with Seahorse XF Calibrant solution in the utility plate (1 ml per well) and incubated in a CO_2_-free incubator at 37 °C overnight. The next day, cell media was replaced with bicarbonate-free XF DMEM assay medium (525 μl per well) (Seahorse Bioscience) supplemented with 25 mM glucose and 2 mM pyruvate, and incubated for 1 h in the CO2-free incubator. Compounds were loaded into the sensor cartridge ports (75 μl per port) and incubated for 1 h. The loaded sensor cartridge and cell plate were then calibrated and ran on the Seahorse Analyzer to assess the cellular respiration rate.

### Functional in vivo mouse studies

The research conducted conformed to the Animals (Scientific Procedures) Act 1986 Amendment Regulations 2012 following ethical review approved by the University of Cambridge Animal Welfare and Ethical Review Body (AWERB). The study is reported in accordance with ARRIVE guidelines. The mice were euthanized by cervical dislocation immediately followed by exsanguination in accordance to the code of Practice for the Humane Killing of Animals under Schedule 1 to the Animals (Scientific Procedures) Act 1986. Enzymatic assay kits were used for determination of serum free fatty acid, triglyceride and 3-hydroxybutyrate concentrations and ELISA kits were used for the measurement of serum insulin, leptin and adiponectin levels, as previously described^50^. Oxygen consumption (VO_2_) and carbon dioxide production (VCO_2_) were measured using an open circuit calorimetry system (Oxymax; Columbus Instruments International, Columbus, OH). Measurements of VO_2_ and VCO_2_ and calculation of RER and EE were as described previously^50^. Body fat mass and lean mass measured by time-domain nuclear magnetic resonance (TD-NMR) by using a minispec Live Mice Analyzer LF50 (Bruker). Glucose tolerance tests and glucose measurements were carried out as previously described^51^.

### Determination of NMY1009 in vivo toxicity

7 treatment groups and 1 vehicle group (four mice per each group) were subjected to 7 daily administrations of the drug i.p. NMY1009 was dissolved in 100% DMSO and the injection volume was 30 μl. Well-being checks were carried out at 6 and 24 h after each treatment. The in vivo toxicity study was carried out by Altogen Labs (Austin, TX).

### Determination of the in vitro metabolic stability of NMY1009 and FCCP upon incubation with rat liver microsomes

NMY1009 and FCCP were detected by LC–MS/MS using the Agilent 1290 Infinity HPLC system (Agilent Technology, Waldbronn, Germany) coupled to a Q Trap™ 5500 hybrid triple quadrupole linear ion trap mass spectrometer (Applied Biosystems/MDS Sciex, Concord, Ontario, Canada). Data processing was performed with Analyst™ 1.5.2 software package (Applied Biosystems, MA., USA). Chromatographic separation was performed on an Zorbax Eclipse Plus C18 column (2.1X 100 mm, i.d., 3.5 µm, Agilent Technologies, Palo Alto, CA, USA) with a Security Guard Cartridge (3.0 X 4 mm, Agilent Technologies, Palo Alto, CA, USA). The mobile phase consisted of acetonitrile containing 5 mM ammonium acetate and water containing 5 mM ammonium acetate (for FCCP, ammonium acetate was substituted with 0.1% formic acid). The flow rate was set at 0.4 ml/min at ambient column temperature. The mass spectrometer was operated using ESI source in negative ion detection mode.

Liver microsomal incubations were conducted in triplicate. Incubation mixtures consisted of 7.5 µl of 20 mg/ml of female or male rat liver microsomes (FRLM and MRLM, respectively, obtained from BD Gentest Corp. (Woburn, MA, USA), final concentration of 0.3 mg microsome protein/mL), 2.5 µl of 200 µM NMY1009 or FCCP in acetonitrile (final concentration of 1 µM), 440 µl of 0.1 M phosphate buffer (pH 7.4). The mixture was pre-incubated for 5 min in a shaking water bath at 37 °C. The reaction was initiated by adding 50 µl of 10 mM NADPH to obtain a final concentration of 1mM NADPH. Aliquots of 50 µl of the incubation sample mixture were collected at 0, 5, 10, 15, 30, and 45 min. After collection of samples, the reaction was terminated with 100 µl of chilled acetonitrile containing the internal standard (0.3 µM FCCP for the quantification of NMY1009; 0.6 µM niclosamide for the quantification of FCCP). The mixture was then centrifuged at 10,000 × g for 10 min to remove the protein and the supernatant was subsequently subjected to LC–MS/MS analysis. The positive control (PC), the known P450 substrate midazolam (3 µM) was treated in the same manner as NMY1009 and FCCP. The negative controls consisted of NMY1009 or FCCP as described above with NADPH omitted in the incubation.

To calculate the metabolic stability, the peak areas of drug were converted to parent remaining percentages, using the t = 0 peak area values as 100%. The remaining percentages of this candidate were plotted against the microsomal incubation time. Data points were the average of three measurements with standard deviations as the error bars.

### Determination of stability of NMY1009 and FCCP in aqueous buffer

The quantification of NMY1009 and FCCP by by LC–MS/MS was similar to the microsomal stability assays, except that the final drug concentration was 10 µM and that myriocin was used as internal standard and mass spectrometry analysis of NMY1009 and FCCP was carried out in positive electrospray ionization (ESI) mode. The calibration curves of the peak area ratio over the analyte concentration gave correlation coefficients r = 0.996 and r = 0.990 for FCCP and NMY1009, respectively. To measure the stability of NMY1009 and FCCP and determine the percentages of the remaining NMY1009 and FCCP after incubation in aqueous 100 mM KPO_4_ buffer, reaction mixtures were quenched with equal volume of acetonitrile at the respective time points. The samples were centrifuged at 4 °C, 4000 rpm for 30 min and 100 μl of the supernatant was used for drug concentration analysis by LC–MS/MS.

### Extraction of NMY1009 from mouse tissues

50 mg of tissue were extracted into 1000 μl of acetonitrile. The entire supernatant was dried and reconstituted into 200 μl of 1:1 acetonitrile/water.

### Measurement of 2,6-dinitrophenol and 2,6-dinitro-4-(octyloxy)phenol tissue and plasma concentrations

In the sample preparation procedure, 50 µl of sample was mixed with 200 µl acetonitrile containing the internal standard telmisartan at a concentration of 200 ng/ml, vortexed for 5 min and centrifuged at 4000 rpm for 5 min at 4 °C. 110 µl of supernatant was separated and diluted with 130 µl of methanol:water(1:1,v/v).

2,6-dinitrophenol and 2,6-dinitro-4-(octyloxy)phenol were detected with the SCIEX QTRAP 5500 + LC–MS/MS system. Data processing was performed with Analyst™ 1.5.2 software package (Applied Biosystems, MA., USA). Chromatographic separation was performed on an Kinetex EVO C18 column. The mobile phase consisted of acetonirile:methanol (50:50,v/v) and 10 mM ammonium acetate with 0.1% formic acid in water. The flow rate was set at 1 ml/min at a column oven temperature of 40 °C. The mass spectrometer was operated using ESI source in the negative ion detection mode.

The measurements of 2,6-dinitrophenol and 2,6-dinitro-4-(octyloxy)phenol tissue and plasma concentrations was carried out by GVK Biosciences (Hyderabad, India).

### Statistical analysis

Statistical analyses were carried out using an unpaired Student’s t-test. EC50 values of uncoupling activities for the various compounds were calculated based on the dose response data using nonlinear regression in GraphPad Prism software.

## Data Availability

The majority of data generated or analyzed during this study are included in this published article. Other primary data (e.g. oxygen consumption tracings) are available from the corresponding author on reasonable request.

## References

[1] Haslam DW, James WP (2005). Obesity. Lancet.

[2] Bray GA (2002). The underlying basis for obesity: Relationship to cancer. J. Nutr..

[3] Pilitsi E (2019). Pharmacotherapy of obesity: Available medications and drugs under investigation. Metabolism.

[4] Kaplan LM (2005). Pharmacological therapies for obesity. Gastroenterol. Clin. North Am..

[5] Müller TD, Clemmensen C, Finan B, DiMarchi RD, Tschöp MH (2018). Anti-obesity therapy: From rainbow pills to polyagonists. Pharmacol. Rev..

[6] Paccosi S, Cresci B, Pala L, Rotella CM, Parenti A (2020). Obesity therapy: How and why?. Curr. Med. Chem..

[7] Wing RR, Phelan S (2005). Long-term weight loss maintenance. Am. J. Clin. Nutr..

[8] Melnikova I, Wages D (2006). Anti-obesity therapies. Nat. Rev. Drug Discov..

[9] Yanovski SZ, Yanovski JA (2002). Obesity. N. Engl. J. Med..

[10] Yanovski SZ, Yanovski JA (2014). Long-term drug treatment for obesity: A systematic and clinical review. JAMA.

[11] Rodgers RJ, Tschöp MH, Wilding JP (2012). Anti-obesity drugs: past, present and future. Dis. Model Mech..

[12] Leibel RL, Rosenbaum M, Hirsch J (1995). Changes in energy expenditure resulting from altered body weight. N. Engl. J. Med..

[13] Chow CC, Hall KD (2008). The dynamics of human body weight change. PLoS Comput. Biol..

[14] Redman LM (2009). Metabolic and behavioral compensations in response to caloric restriction: implications for the maintenance of weight loss. PLoS One.

[15] Kaplan LM (2010). Pharmacological therapies for obesity. Gastroenterol. Clin. North Am..

[16] Terada H (1990). Uncouplers of oxidative phosphorylation. Environ. Health Perspect..

[17] Parker VH (1958). Effect of nitrophenols and halogenophenols on the enzymic activity of rat-liver mitochondria. Biochem. J..

[18] Hemker HC (1962). Lipid solubility as a factor influencing the activity of uncoupling phenols. Biochim. Biophys. Acta.

[19] Matsuno-Yagi A, Hatefi Y (1989). Uncoupling of oxidative phosphorylation: Different effects of lipophilic weak acids and electrogenic ionophores on the kinetics of ATP synthesis. Biochemistry.

[20] Harper ME, Green K, Brand MD (2008). The efficiency of cellular energy transduction and its implications for obesity. Annu. Rev. Nutr..

[21] Horner WD, Jones RB, Boardman WW (1935). Cataracts following the use of dinitrophenol, preliminary report of 3 cases. JAMA.

[22] Horner WD (1936). Cataract following dinitrophenol treatment for obesity. Arch. Ophthal..

[23] Ogino S, Yasukura K (1957). Biochemical studies on cataract. VI. Production of cataracts in guinea pigs with dinitrophenol. Am. J. Ophthalmol..

[24] Colman E (2007). Dinitrophenol and obesity: An early twentieth-century regulatory dilemma. Regul. Toxicol. Pharmacol..

[25] Murray JH (2023). Oxadiazolopyridine derivatives as efficacious mitochondrial uncouplers in the prevention of diet-induced obesity. J. Med. Chem..

[26] Caldeira da Silva CC, Cerqueira FM, Barbosa LF, Medeiros MHG, Kowaltowski AJ (2008). Mild mitochondrial uncoupling in mice affects energy metabolism, redox balance, and longevity. Aging Cell.

[27] Goldgof M, Xiao C, Chanturiya T, Jou W, Gavrilova O, Reitman ML (2014). The chemical uncoupler 2,4-dinitrophenol (DNP) protects against diet-induced obesity and improves energy homeostasis in mice at thermoneutrality. J. Biol. Chem..

[28] Perry RJ (2013). Reversal of hypertriglyceridemia, fatty liver disease, and insulin resistance by a liver-targeted mitochondrial uncoupler. Cell Metab..

[29] Liang S (2023). Non-cardiotoxic tetradecanoic acid-2,4-dinitrophenol ester nanomicelles in microneedles exert potent anti-obesity effect by regulating adipocyte browning and lipogenesis. Small.

[30] Drobnica L, Sturdík E (1979). The reaction of carbonyl cyanide phenylhydrazones with thiols. Biochim. Biophys. Acta.

[31] Mlejnek P, Dolezel P (2015). Loss of mitochondrial transmembrane potential and glutathione depletion are not sufficient to account for induction of apoptosis by carbonyl cyanide 4 (trifluoromethoxy)phenylhydrazone in human leukemia K562 cells. Chem. Biol. Interact..

[32] Khailova LS, Firsov AM, Kotova EA, Antonenko YN (2019). Interaction of potent mitochondrial uncouplers with thiol-containing antioxidants. Antioxidants (Basel).

[33] Liberman EA, Topaly VP, Tsofina LM, Jasaitis AA, Skulachev VP (1969). Mechanism of coupling of oxidative phosphorylation and the membrane potential of mitochondria. Nature.

[34] Geyer HJ, Scheunert I, Rapp K, Gebefügi I, Steinberg C, Kettrup A (1993). The relevance of fat content in toxicity of lipophilic chemicals to terrestrial animals with special reference to dieldrin and 2,3,7,8-tetrachlorodibenzo-p-dioxin (TCDD). Ecotoxicol. Environ. Saf..

[35] Müllerová D, Kopecký J (2007). White adipose tissue: Storage and effector site for environmental pollutants. Physiol. Res..

[36] Jackson E, Shoemaker R, Larian N, Cassis L (2017). Adipose tissue as a site of toxin accumulation. Compr. Physiol..

[37] Morstein J (2022). Medium-chain lipid conjugation facilitates cell-permeability and bioactivity. J. Am. Chem. Soc..

[38] Hemker HC, Hülsmann WC (1961). Dinitrophenol-induced ATPase of rat-liver mitochondria. Biochim. Biophys. Acta.

[39] Denisov SS, Kotova EA, Khailova LS, Korshunova GA, Antonenko YN (2014). Tuning the hydrophobicity overcomes unfavorable deprotonation making octylamino-substituted 7-nitrobenz-2-oxa-1,3-diazole (n-octylamino-NBD) a protonophore and uncoupler of oxidative phosphorylation in mitochondria. Bioelectrochemistry.

[40] Shchepinova MM (2014). Dodecyl and octyl esters of fluorescein as protonophores and uncouplers of oxidative phosphorylation in mitochondria at submicromolar concentrations. Biochim. Biophys. Acta.

[41] Castagnetti E, Schlosser M (2002). The trifluoromethoxy group- A long-range electron-withdrawing substituent. Chemistry.

[42] Leroux FR, Manteau B, Vors JP, Pazenok S (2008). Trifluoromethyl ethers – synthesis and properties of an unusual substituent. Beilstein J. Org. Chem..

[43] Inoue Y, Wada Y, Sato M, Sato S, Okamoto T, Kanemoto N (2023). Carbonyl cyanide-4-(trifluoromethoxy)phenylhydrazone-induced toxicities in rats: Comparative study with other mitochondrial uncouplers (2,4-dinitrophenol, OPC-163493 and tolcapone). Toxicol. Res..

[44] Han YH, Kim SH, Kim SZ, Park WH (2009). Carbonyl cyanidep-(trifluoromethoxy) phenylhydrazone (FCCP) as an O2•−generator induces apoptosis via the depletion of intracellular GSH contents in Calu-6 cells. Lung Cancer.

[45] Han YH, Park WH (2011). Intracellular glutathione levels are involved in carbonyl cyanide p-(trifluoromethoxy) phenylhydrazone-induced apoptosis in As4.1 juxtaglomerular cells. Int. J. Mol. Med..

[46] Kopecky J, Clarke G, Enerbäck S, Spiegelman B, Kozak LP (1995). Expression of the mitochondrial uncoupling protein gene from the aP2 gene promoter prevents genetic obesity. J. Clin. Invest..

[47] Kopecký J, Hodný Z, Rossmeisl M, Syrový I, Kozak LP (1996). Reduction of dietary obesity in aP2-Ucp transgenic mice: physiology and adipose tissue distribution. Am. J. Physiol..

[48] Rossmeisl M (2002). Expression of the uncoupling protein 1 from the aP2 gene promoter stimulates mitochondrial biogenesis in unilocular adipocytes in vivo. Eur. J. Biochem..

[49] Zolfigol MA, Madrakian E, Ghaemi E (2003). Trichloroisocyanuric Acid/NaNO_2_/wet SiO_2_ as an efficient system for the selective dinitration of phenols under solvent-free conditions. Synlett.

[50] Lelliott CJ (2006). Ablation of PGC-1b results in defective mitochondrial activity, thermogenesis, hepatic function, and cardiac performance. PLoS Biol..

[51] Medina-Gomez G (2005). The link between nutritional status and insulin sensitivity is dependent on the adipocyte-specific peroxisome proliferator-activated receptor-gamma2 isoform. Diabetes.

